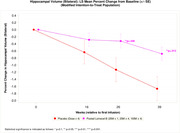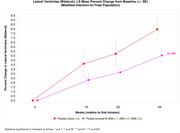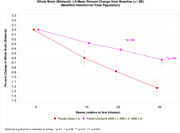# Reduction in Alzheimer’s disease brain atrophy by MRI measures in a phase 2a clinical trial of Lomecel‐B^TM^

**DOI:** 10.1002/alz.092494

**Published:** 2025-01-09

**Authors:** Kevin N. Ramdas, Nataliya Agafonova, Joshua M. Hare, Eric Naioti, Steven Kopcho, Raul Carballosa, Paayal Patel, Mark Brody, Brad Herskowitz, Ana Fuquay, Savannah Rodriguez, Jeffrey Botbyl, Brian Rash, Anthony A. Oliva

**Affiliations:** ^1^ Longeveron Inc., Miami, FL USA; ^2^ First Excellent Research, Miami, FL USA; ^3^ Brain Matters Research, Delray Beach, FL USA; ^4^ Brainstorm Research, Miami, FL USA

## Abstract

**Background:**

Alzheimer’s disease (AD) is characterized by progressive atrophy of the cerebral cortex and hippocampus, with concomitant increase in ventricular volume. Lomecel‐B is a novel cell‐based therapeutic approach to AD that targets neuroinflammation, microvascular dysfunction, and has the potential to stimulate endogenous tissue regeneration. We conducted MRI analysis of brain morphology in the CLEAR‐MIND study, a 49‐patient proof‐of‐concept study that tested 3 different dosing regimens of Lomecel‐B vs placebo in patients with mild AD dementia.

**Methods:**

This double‐blind, randomized, placebo‐controlled 45‐week proof‐of‐concept trial (ClinicalTrials.gov: NCT05233774) enrolled patients (60‐85 yrs.) with mild AD dementia (MMSE score 18‐24); with evidence of amyloid on positron‐emission tomography (PET) and brain MRI consistent with AD. There were 4 study arms of Lomecel‐B intravenous infusion: 25 million (25M) cells once followed by 3 infusions of placebo (*N* = 13); 25M cells (*N* = 13) or 100 million (100M) cells (*N* = 11) monthly for 4 months; 4 IV infusions of Placebo (*N* = 12). Lomecel‐B treatment groups were evaluated for individual and pooled (25Mx1, 25Mx4, 100Mx4) groups vs placebo, n = 32.

**Results:**

Patients receiving Lomecel‐B exhibited slowing of whole brain atrophy (pooled: reduced by 48% vs placebo, p = 0.005). Lomecel‐B exhibited reduced left, right and bilateral hippocampal atrophy by 62%, p = 0.021, 53%, p = 0.073 and 59%, p = 0.013, respectively. At Week 39, left, right and bilateral ventricular enlargement were reduced in Lomecel‐B pooled group by up to 34%, p = 0.097, 40%, p = 0.044 and 37%, p = 0.066 respectively, compared to placebo. Moreover, diffuse tensor imaging (DTI) used to index neuroinflammation indicated increased inflammation in AD placebo but substantial reduction in the Lomecel‐B pooled group, particularly the cingulate cortex (100% reduction, p = 0.002).

**Conclusion:**

Brain MRI in this proof‐of‐concept study with small sample size reveals that Lomecel‐B was associated with measurable neuroanatomical and neuroinflammatory improvements compared with progressive AD pathology observed in placebo. Accordingly, these findings support further development of Lomecel‐B in a larger dose‐finding study.